# Comparison between minimally invasive percutaneously and open pedicle screw fixation of thoracolumbar fracture: Prospective comparative study protocol

**DOI:** 10.1097/MD.0000000000023403

**Published:** 2020-12-04

**Authors:** Qianhuan Gui, Xiaotao Su, Zhenghao Lu, Jun He

**Affiliations:** Department of Spine Surgery, the Affiliated Nanhua Hospital of University of South China, Hengyang, Hunan, China.

**Keywords:** minimally invasive percutaneous pedicle screw fixation, prospective, study protocol, thoracolumbar fracture

## 1 Introduction

Thoracolumbar fracture is familiar spinal injuries in the high-energy violence and traffic accidents, accounting for more than 50 percent of the total number of spinal fractures.^[1–3]^ Despite some thoracolumbar fractures patients who do not have neurologic dysfunction respond well to conservative treatment, short-segment pedicle screw fixation has been proven to be a more effective approach to resort vertebral height, correct kyphosis and stabilize fractures.^[4,5]^

In the past few years, minimally invasive percutaneous pedicle screw fixation (MIPPSF) as the alternative approach to treat the thoracolumbar fractures, aims to decrease the injury of soft tissue and perioperative complications. Originally used to treat degenerative spine diseases, the MIPPSF system have been demonstrated to be effective in decreasing blood loss and complications, avoiding muscle and tissue injury, and shortening the hospital stays and recovery times.^[6–8]^ Although there are similar advantages in treating the thoracolumbar fractures, the evidence is mostly limited to low-energy and low-level observational studies. In addition, the lack of long-term efficacy of the MIPPSF in treating the thoracolumbar fractures further limits the available evidence for this technique.^[9–13]^

Therefore, the purpose of our study is to compare the radiological and clinical results of thoracolumbar spine injury stabilized by standard open pedicle screw fixation (OPSF) and the MIPPSF system. We hypothesize that MIPPSF technique will lead to better clinical and radiological outcomes compared to OPSF technique.

## 2 Materials and methods

### 2.1 Study design and eligibility criteria

**Figure 1:**
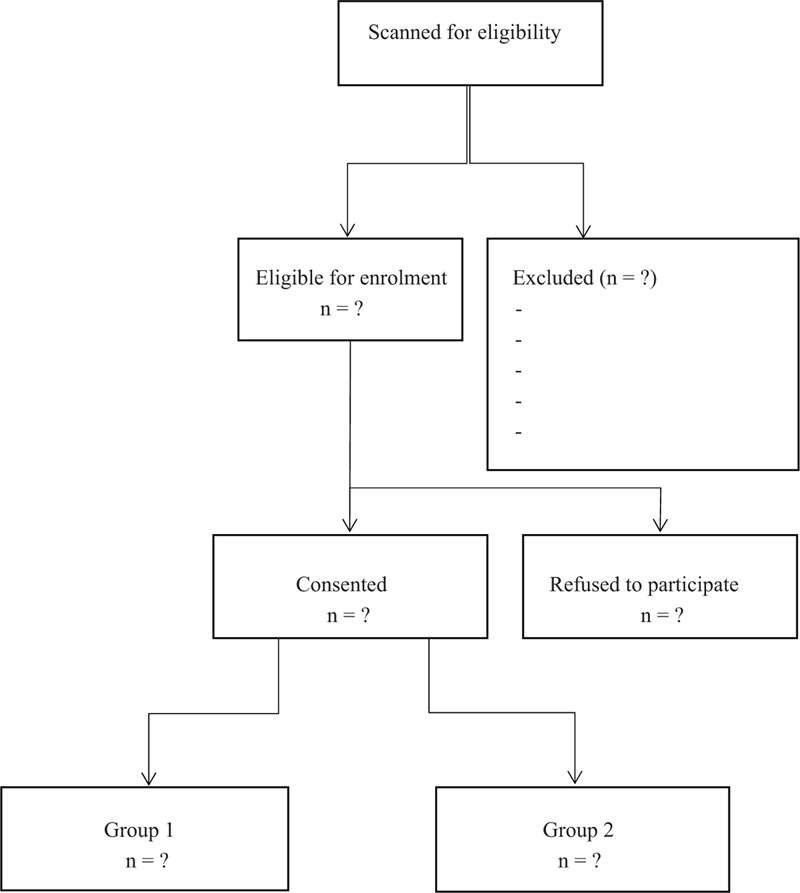
Flow diagram of the study.

This study is designed as a prospective non-randomized cohort study, which will be conducted at our own hospital between November 13, 2020 to November 14, 2021. This study has been granted through the institutional review committee of the Affiliated Nanhua Hospital of University of South China (with the number is NY7002). All the patients participating in the study will give the written informed consent. Afterwards, the scheme of our experiment has been registered with the Research Registry (researchregistry6140). Patients will be alternately included to both groups according to the following inclusion criteria: patients with single level thoracolumbar fracture (T11-L2); Patients who underwent surgery for less than a week after trauma; the absence of neurological deficits and no other significant injury. Patients with pedicle fracture, pathological fracture, severe bone loss, or prior spinal surgery owing to the trauma are excluded from our research. Patients who need the direct decompression of the spinal canal because of neurological deficits are also be excluded (Fig. 1).

### 2.2 Operation procedure

MIPPSF is conducted via adopting a new type of percutaneous pedicle screw system independently developed and improved through our hospital, while OPSF adopts the traditional system of thoracolumbar pedicle screw. All the operations will be implemented via a same senior surgeon. After the general anesthesia, the patients receiving OPSF will be placed in a prone position. A conventional incision is utilized to expose fractured vertebra and its adjacent vertebrae. These vertebrae are fixed with pedicle screws, and then the appropriate distraction reduction is performed. No bone graft spinal fusion is implemented. The MIPPSF patient is anesthetized and then lies prone with the abdomen away from the operating table. Spinal fusion with bone graft is not performed. After the MIPPSF patient is anesthetized, the abdomen leaves the operating table and the patient lies in a prone position. Fractured vertebra is located with C-arm X-ray system. The fascia and skin are cut 1 cm outside the 2 pedicles projections, and then the trocar is inserted, with the trocar tip at 3 o ‘clock and 9 o ‘clock in the right pedicle, respectively. The core needles are removed and the wires are inserted. After the spinal canal is dilated and tapped with the thread tap, the novel independently improved pedicle screws are inserted, and then the titanium rods are inserted. The improved percutaneous pedicle screws possess various bending angles, which is conducive to automatic fracture reduction in the process of traction. Subsequently, while tightening the screw, the surgeon reverses the rotation of the outer nut at the end of the improved screw. Ultimately, C-arm X-ray is used to check the reduction again. The incision is cleaned and then closed, and then the drainage strip is placed.

### 2.3 Clinical and radiologic assessments

The primary clinical outcome in our study is visual analog scale score, the other outcomes are low back outcome score, intraoperative bleeding, length of hospital stay, and complications. The Cobb angle and vertebral height ratio are detected via the imaging data before and after the fracture vertebra. The visual analog scale score, low back outcome score, complications, and radiologic outcomes will be measured at 3, 6, and 12 months.

### 2.4 Sample size calculation

We estimate that when there are 30 participants in each group, our experiment will have more than 80 percent of the ability to determine the clinically significant differences between the 2 groups in terms of changes in the scores of pain assessed by visual analog scale score. This assumes that, on the basis of former literature, the mean difference in scores between groups is 20 mm, the combined standard deviation is 35 mm based on the preliminary data, and the alpha level is 5%. According to this estimate, a total of 66 patients will be required and a 10% drop-out allowance will be given.

### 2.5 Statistical analysis

The software of SPSS v22.0 is utilized to implement the statistical analyses (IBM, Chicago, IL). The comparison of demographic characteristics between the 2 groups, follow-up time and clinical hip scores are carried out by descriptive statistics. The statistical analysis contained Fisher exact test for the categorical variables and Student *t* test for the continuous variables. All the values of probability were 2-tailed, and when the *P* value less then .05, it can be viewed as the statistically significant.

## 3 Discussion

The major purpose of our study is to compare the radiological and clinical results of thoracolumbar spine injury stabilized by standard OPSF and the MIPPSF system. We hypothesize that MIPPSF technique will lead to better clinical and radiological outcomes compared to OPSF technique. The biggest limitations of this study is non-randomized design, because we cannot obtain the approval of the Ethics Committee for randomized research, so even if the 2 groups of patients are comparable, it may cause the biased choice of patients. The in-depth randomized studies in this direction may make the application of this procedure clearer.

## Author contributions

**Conceptualization:** Qianhuan Gui, Jun He, Zhenghao Lu.

**Data curation:** Qianhuan Gui, Xiaotao Su.

**Formal analysis:** Qianhuan Gui, Xiaotao Su.

**Funding acquisition:** Jun He.

**Investigation:** Qianhuan Gui, Xiaotao Su.

**Methodology:** Xiaotao Su, Jun He.

**Project administration:** Jun He.

**Resources:** Zhenghao Lu, Jun He.

**Software:** Zhenghao Lu, Xiaotao Su.

**Supervision:** Jun He.

**Validation:** Qianhuan Gui, Zhenghao Lu.

**Visualization:** Xiaotao Su, Zhenghao Lu.

**Writing – original draft:** Qianhuan Gui.

**Writing – review & editing:** Zhenghao Lu, Jun He.
